# Heterogeneous genetic patterns in bilateral perisylvian polymicrogyria: insights from a Finnish family cohort

**DOI:** 10.1093/braincomms/fcae142

**Published:** 2024-04-18

**Authors:** Irma Järvelä, Ritva Paetau, Yasmin Rajendran, Anushree Acharya, Thashi Bharadwaj, Suzanne M Leal, Anna-Elina Lehesjoki, Maarit Palomäki, Isabelle Schrauwen

**Affiliations:** Department of Medical Genetics, University of Helsinki, 00251 Helsinki, Finland; Department of Child Neurology, University of Helsinki and Helsinki University Hospital, 00290 Helsinki, Finland; Center for Statistical Genetics, Gertrude H. Sergievsky Center, Department of Neurology, Columbia University Medical Center, 10032 New York, NY, USA; Center for Statistical Genetics, Gertrude H. Sergievsky Center, Department of Neurology, Columbia University Medical Center, 10032 New York, NY, USA; Center for Statistical Genetics, Gertrude H. Sergievsky Center, Department of Neurology, Columbia University Medical Center, 10032 New York, NY, USA; Center for Statistical Genetics, Gertrude H. Sergievsky Center, Department of Neurology, Columbia University Medical Center, 10032 New York, NY, USA; Taub Institute, Columbia University Medical Center, 10032 New York, NY, USA; Department of Medical Genetics, University of Helsinki, 00251 Helsinki, Finland; Folkhälsan Research Center, 00290 Helsinki, Finland; Medical Imaging Center, University of Helsinki and Helsinki University Hospital, 00290 Helsinki, Finland; Center for Statistical Genetics, Gertrude H. Sergievsky Center, Department of Neurology, Columbia University Medical Center, 10032 New York, NY, USA

**Keywords:** biparietal perisylvian polymicrogyria, exome sequencing, gene, *de novo*, optical genome mapping

## Abstract

Bilateral perisylvian polymicrogyria is the most common form of regional polymicrogyria within malformations of cortical development, constituting 20% of all malformations of cortical development. Bilateral perisylvian polymicrogyria is characterized by an excessive folding of the cerebral cortex and abnormal cortical layering. Notable clinical features include upper motoneuron dysfunction, dysarthria and asymmetric quadriparesis. Cognitive impairment and epilepsy are frequently observed. To identify genetic variants underlying bilateral perisylvian polymicrogyria in Finland, we examined 21 families using standard exome sequencing, complemented by optical genome mapping and/or deep exome sequencing. Pathogenic or likely pathogenic variants were identified in 5/21 (24%) of families, of which all were confirmed as *de novo.* These variants were identified in five genes, i.e. *DDX23*, *NUS1*, *SCN3A*, *TUBA1A* and *TUBB2B*, with *NUS1* and *DDX23* being associated with bilateral perisylvian polymicrogyria for the first time. In conclusion, our results confirm the previously reported genetic heterogeneity of bilateral perisylvian polymicrogyria and underscore the necessity of more advanced methods to elucidate the genetic background of bilateral perisylvian polymicrogyria.

## Teaser

Järvelä *et al*. report *de novo* pathogenic or likely pathogenic variants in five genes underlying bilateral perisylvian polymicrogyria in a Finnish cohort using exome sequencing, optical genome mapping and deep sequencing. The outcome identified variants in 24% of families, expanding the genetic heterogeneity of bilateral perisylvian polymicrogyria.

## Graphical Abstract

**Graphical Abstract:**
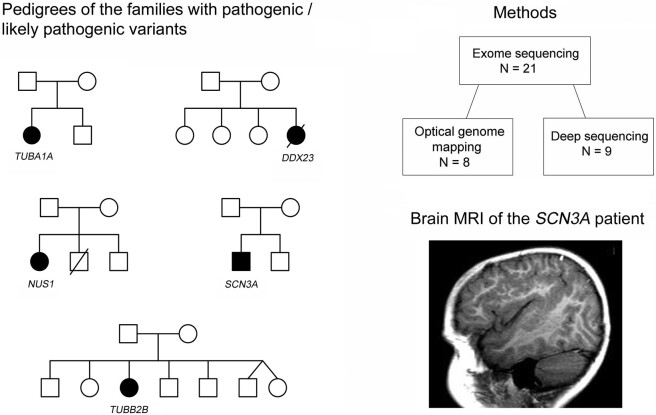

## Introduction

Malformations of cortical development (MCD) are rare congenital anomalies of the cerebral cortex falling under neuronal migration defects.^1^ MCDs are classified into three major groups: malformations caused by abnormal cell proliferation, misdirected neuronal migration, or aberrant post-migrational cortical organization and connectivity.^1,2^ Polymicrogyria (PMG) is the most common form accounting for 20% of all MCDs.^3^ PMG is characterized by an overfolding of the cerebral cortex and abnormal cortical layering, which is diagnosed by brain imaging such as MRI. Both genetic (non-syndromic and syndromic forms) and non-genetic causes have been reported for PMG.^4^ Currently, ∼50 genes have been identified as responsible for PMGs, accounting for ∼20–32% of the cases.^4-8^

Bilateral perisylvian polymicrogyria (BPP) is the most common type of regional PMG.^9^ The major clinical features include upper motor neuron dysfunction, dysarthria and asymmetric quadriparesis. Cognitive impairment and different types of epilepsy have also been described. The clinical features in PMG, and even in BPP, can be quite variable and heterogeneous.

For BPP, Mirzaa *et al*.^5^ identified a recurrent mosaic variant (p.Gly373Arg) in *PIK3R2*. We previously discovered a pathogenic variant in *SCN3A* that underlies a unique neurodevelopmental channelopathy in a Finnish BPP cohort.^8^ In this study, we used a stepwise approach of standard exome sequencing (ES), followed by deep ES sequencing of buccal-derived DNA (ectoderm lineage) and/or optical mapping (OGM) to find genetic causes for 21 families in a Finnish BPP cohort.

## Materials and methods

### Study approval

Written informed consent was obtained from the parents and the patients according to the Declaration of Helsinki. Written informed consent was obtained to publish photographs of the patients. The study was approved by the ethics committees of the Hospital District of Helsinki and Uusimaa (HUS/2532/2017) and the Institutional Review Boards of Columbia University (IRB-AAAS3433).

### Family ascertainment and phenotype analysis

A total of 21 Finnish families with 22 cases of BPP detected by MRI were enrolled in the study. The samples were collected between 1998 and 2023 and included 18 trios, 2 duos and 1 singleton case. Affected individuals were clinically evaluated by one of the authors (R.P.). None of the individuals had extrinsic non-genetic causes, including vascular, hypoxic insults or congenital cytomegalovirus infection. Peripheral blood samples were collected from study participants that included both affected and unaffected family members. Genomic DNA was isolated following standard procedures. Families with inconclusive/negative exome results were studied further via deep buccal sequencing and/or OGM. Unfortunately, we were unable to collect a buccal sample and/or an additional blood sample for certain negative/inconclusive families (Supplementary Table 1). Ultra-high-molecular-weight (UHMW) DNA from frozen peripheral blood samples of eight individuals was extracted using the Prep SP Blood and Cell Culture DNA Isolation Kit (Bionano Genomics, San Diego, CA, USA). Buccal epithelial cells were collected from nine affected individuals. Buccal DNA was isolated using the prepIT L2P extraction kit (DNA Genotek Inc., Ottawa, ON, Canada).

### ES and bioinformatic analysis

Exomic libraries were prepared using the SureSelect Human All Exon V6 kit (Agilent Technologies, Santa Clara, CA, USA). Paired-end sequencing was performed on a NovaSeq6000 instrument (Illumina Inc., San Diego, CA, USA), targeting 50× for standard ES (using blood-derived DNA) and 200× for deep ES (using buccal-derived DNA). Reads were aligned to the human genome (hg38) using Burrows–Wheeler alignment-maximal exact match (MEM),^10^ and single nucleotide variants (SNVs) and small insertion/deletions (indels) were called following the Genome Analysis Toolkit best practices in variant calling.^11^ Copy number variants (CNVs) were called using Copy Number Inference from Exome Reads^12^ (PMID: 22585873).

Rare variants following several inheritance models (e.g. *de novo*, autosomal recessive, autosomal dominant and X-linked) with a predicted effect on protein function or pre-mRNA splicing were retained. We also performed an additional analysis to detect variants present only in the ectodermal lineage (buccal). In this case, standard and deep exome sequences of the same cases were called jointly, and variants present in the standard exome sequence data were excluded.

Sanger sequencing was performed using an ABI3130XL Genetic Analyzer to verify candidate SNV and InDel variants and to examine segregation among the family members that were not exome-sequenced. The classification of variants is based on the American College of Medical Genetics and Genomics recommendations.^13^

### Optical genome mapping and bioinformatic analysis

UHMW DNA labelling was processed with the Bionano Prep DLS DNA Labeling kit (Bionano Genomics). Labelled DNA was loaded on a Saphyr chip and run on a Saphyr instrument (Bionano Genomics), targeting ∼200× coverage. A *de novo* genome map assembly was performed using Bionano Solve™, followed by structural variant calling. Data were analysed with Bionano Access™ and was further annotated with annotSV^14^ and biomaRt.^15^ Rare variants, based on their frequency in the Database of Genomic Variants, dbVar (NCBI), gnomAD and 279 controls from Bionano Genomics, were prioritized.

## Results

**Figure 1:**
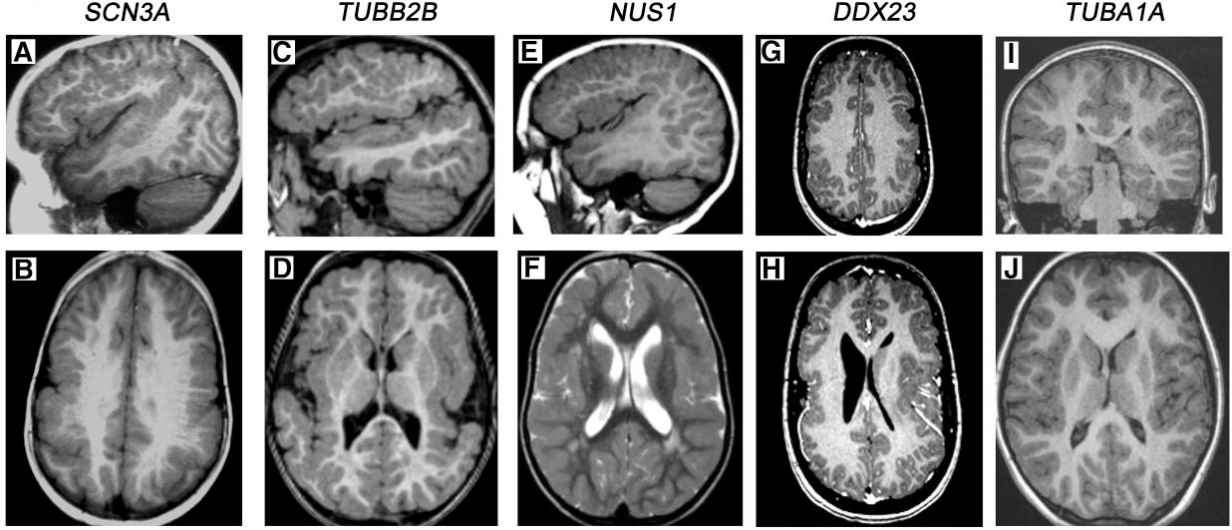
**Brain MR images of patients with pathogenic or likely pathogenic gene variants.** The subject with an SCN3A variant shows BPP and thick grey matter over the frontal, parietal and perisylvian cortexes (**A, B**). The subject with a TUBB2B variant has bilateral fronto-parietal, perisylvian and insular polymicrogyria (**C, D**). The subject with an NUS1 variant has a similar pattern of distribution of polymicrogyria as seen with SCN3A and TUBB2B but additional white matter changes around the posterior horn of the lateral ventricles (**E, F**). The subject with a DDX23 variant has asymmetric patches of frontal, parietal and insular/perisylvian polymicrogyria (**G, H**). The polymicrogyric cortex of the subject with a TUBA1A variant is limited to the opercular and insular cortexes (**I, J**). SCN3A, the sodium voltage–gated channel alpha subunit 3; TUBB2B, tubulin 2B; NUS1, dehydrodolichyl diphosphate synthase subunit; *DDX23*, DEAD-box RNA helicase 23 gene; TUBA1A, tubulin alpha-1A.

We found novel pathogenic (P)/likely pathogenic (LP) variants in five genes known to underlie a neurodevelopmental disorder in 21 (24%) families with BPP using ES (Table 1; Supplementary Materials). All of them were *de novo.* Their brain MRI images are presented in Fig. 1. Detailed clinical information is given in Supplementary Materials.

**Table 1:** Identification of pathogenic and likely pathogenic variants in BPP

Patient *#*	Sex	Age	Gene	Variant	Inheritance	Severity of ID or DD	MRI/CT	Other features	ACMG	Evidence
FINLIS2	M	18	*SCN3A*	NM_006922.4:c.4862G>A:p.(R1621Q)	*De novo*	Mild	BPP	Epilepsy	P	PS2, PM2, PP2, PS1, PS3
FINLIS4	F	22	*TUBB2B*	NM_178012.5:c.776C>T:p.(P259L)	*De novo*	Mild	BPP	Epilepsy	P	PS2, PP2, PM2, PS1_sup, PM1
FINLIS14	F	14	*NUS1*	NM_138459.5:c.616G>T:p.(D206Y)	*De novo*	Mild	BPP	Hearing impairment	LP	PS2, PM2
FINLIS18	F	19^a^	*DDX23*	NM_004818.3:c.2437C>T:p.(R813C)	*De novo*	ND	BPP	Rhabdomyosarcoma	LP	PS2, PM2, PP2
FINLIS22	F	11	*TUBA1A*	NM_006009.4:c.303T>A:p.(N101K)	*De novo*	Moderate	Insular cortex	Spastic diplegia	P	PS2, PP3, PM2, PM1, PP2, PM5
*Other identified pathogenic and likely pathogenic variants*										
FINLIS1	M	34	*WFS1*	NM_006005.3:c.923C>G:p.(S308C)^b^	AD	Borderline	BPP	Wolfram-like syndrome	LP	PP1_sup, PP3, PM2, PS1

^a^Deceased.

^b^This variant explains only the hearing loss seen in the mother and child. The cause of BPP in the affected child remains unknown.

In FINLIS2-3, a *de novo* variant p.(Arg1621Gln) in the *sodium voltage–gated channel alpha subunit 3 (SCN3A)* gene (OMIM # 617935) was identified. He presented with severe oral motor dysfunction and autonomic seizures from infancy. At 15 years, he experienced tonic–clonic seizures, but these were ameliorated with valproate and oxcarbazepine treatment. He has mild intellectual disability (ID). A brain MRI identified BPP.

FINLIS4-3 had a *de novo* missense variant p.(Pro259Leu) in *tubulin 2B (TUBB2B)* (OMIM # 610031). Her phenotype was characterized by mild right-dominant tetraplegia, severe dysarthria, nocturnal epileptic myoclonus, mild ID and bilateral perisylvian and perirolandic PMG.

FINLIS14-3 had a *de novo* missense p.(Asp206Tyr) variant in the *dehydrodolichyl diphosphate synthase subunit* (*NUS1*; OMIM # 617831). The phenotype was characterized by mild right-hand paresis, severe oral-motor and oral sensory problems, severe congenital sensorineural deafness (diagnosed at 2 years of age) and mild ID. She has no epilepsy or dysmorphism. Her social skills are good but challenged by anxiety in daily activities. Her MRI displayed BPP and bilateral white matter changes around the posterior horns of the lateral ventricles.

FINLIS18-3 had a *de novo* missense variant p.(Arg813Cys) in the *DEAD-box RNA helicase 23 gene* (*DDX23*; OMIM # 612172). The phenotype was severe, including problems of swallowing with aspiration that led to tube feeding and permanent tracheostomy as a newborn. Permanent gastrostomy for feeding was inserted at 2.5 years of age. Due to the hypoplastic lower jaw, the tongue and the epiglottis and the paralysed tongue and pharyngeal muscles, she could not swallow. She learnt to walk from 3 years of age but never learnt to produce any vocalization. At 7 years of age, her neuropsychological profile showed normal performance. A brain MRI at ages 4 and 8 showed BPP. At 16 years of age, an aggressive rhabdomyosarcoma appeared in the left cheek, resistant to radiation and chemotherapies that invaded large areas of the neck and extra-cranial structures, and finally, the left hemisphere. She passed away at 19 years of age.

FINLIS22-3 has a *de novo* missense variant in the N-terminal region in Exon 3 (p.Asn101Lys) of *tubulin alpha-1A* (*TUBA1A)*, a well-known gene for MCDs.^16^ Her oral motor function is close to normal, whilst spastic diplegia and moderate ID dominate the phenotype. A brain MRI demonstrated that BPP is mainly located in the insular cortex.

The underlying cause of BPP was not identified for the proband in FINLIS1. However, in addition to BPP, the affected son and his unaffected mother have hearing impairment. We identified a previously reported^17^ missense variant p.(Ser308Cys) in *Wolframin ER Transmembrane Glycoprotein* (*WFS1)* underlying autosomal-dominant Wolfram-like syndrome (#OMIM 614296) in both the mother and the son. This agrees with the segregation of autosomal-dominant hearing impairment in this family (data not available) but does not explain BPP.

ES revealed variants of unknown significance in several families (Supplementary Table 2; Supplementary Materials). Deep ES of buccal epithelial DNA was utilized in nine inconclusive/negative cases, where a sample could be obtained. No disease-causing variants were identified (Supplementary Tables 1 and 2). Eight subjects, with available frozen blood samples, whose ES did not reveal a relevant candidate gene, were analysed using OGM, but no variants were identified (Supplementary Table 1). In addition, no CNVs were found through ES.

## Discussion

We applied standard ES, followed by deep ES of buccal samples and/or OGM to identify the genetic causes of BPP. We found pathogenic or likely pathogenic variants in 24% of the analysed families, all of which were *de novo*. The results are similar to those of previous studies.^4,7^ In addition to identifying variants in well-known BPP genes, i.e. *TUBA1A*, *TUBB2B* and *SCN3A*,^6,8^ we propose *DDX23*, a member of the *RNA-helicase* gene family,^18^ and *NUS1*^19^ as novel genes underlying BPP, expanding their phenotypic spectrum.

Interestingly, Zaman *et al*.^20^ reported the same variant p.(Arg1621Gln) in *SCN3A* identified by us in a patient with severe ID, and normal EEG and a different variant in the same codon p.(Arg1621Gly), in a patient with severe ID and pseudobulbar palsy. We previously reported a different variant p.(Phe1759Tyr) in *SCN3A* in a large Finnish family with BPP.^8^ The cognition of the affected individuals ranged from mild ID to borderline intelligence.^8^

*TUBA1A* and *TUBB2B* belong to *tubulinopathies* associated with a wide range of MCDs.^17,21,22^ Among individuals with PMG, including the most common perisylvian subtype, *TUBA1A* variants have been found in 3.1% of all patients with PMG and in 10% of PMG with complex cerebral malformations.^17^ Pathogenic *TUBB2B* variants have been estimated to be involved in 2.3% of MCDs.^23^ The clinical phenotypes caused by the variants of *TUBA1A* can vary considerably; most affected patients have congenital microcephaly and severe neurodevelopmental delay with di/tetraplegia.^1^ FINLIS22-3 had BPP mainly located in the insular cortex. She had normocephaly, a relatively mild upper motor neuron dysfunction and no epilepsy. *TUBA1A* variants were found as the most common cause of microlissencephalies in foetuses.^22^ The missense variant p.(Asn101Lys) in *TUBA1A* in FINLIS22-3 was located in the same codon as previously described in a foetus with p.(Asn101Ser) that showed a poorly differentiated cortical plate^22^ and in a person with microlissencephaly.^21^ FINLIS4-3 with a pathogenic missense variant p.(Pro259Leu) in *TUBB2B* had bilateral perisylvian and perirolandic PMG. Previously, *TUBB2B* variants have been reported to underlie more extensive brain malformations and severe phenotypes.^24^ Of note, patients with *tubulinopathy* in our study had a normal corpus callosum, brainstem and cerebellum. Our results further strengthen the role of *tubulin* genes in BPP and suggest further heterogeneity both in clinical features and in MRI findings.

We propose phenotypic expansions of two known disease genes, *NUS1* and *DDX23*, to include BPP. *NUS1* variants are associated with a wide spectrum of phenotypes.^25^ In addition to the characteristic features of BPP (mild right-hand paresis, severe oral-motor problems and oral sensory problems), a patient in this study had severe congenital sensorineural deafness. In her ES, we did not find a separate variant in a gene that would explain her deafness. However, hearing impairment is seen in patients with autosomal recessive *NUS1* (a congenital disorder of glycosylation; OMIM # 617082).^26^

*DDX23* belongs to the *DExD/H-box RNA helicase g*enes that have been associated with multi-organ diseases, neurodevelopmental disorders, brain malformations and several types of cancer.^18,27,28^  *DDX23* (OMIM #612172) has recently been reported to be associated with a novel syndromic neurodevelopmental disorder.^29^ The phenotype includes developmental delay, muscle tone abnormalities and dysmorphic features. Brain abnormalities, including decreased white matter, corpus callosum abnormalities and grey matter heterotopia, have been noted in some patients.^29,30^ The variant p.(Arg813Cys) is located near the C-terminal RecA-like domain at the 3′ end of the *DDX23* gene at a highly conserved amino acid (GERP_RS_: 5.97) and may explain the severe phenotype of FINLIS18-3. Yin *et al*.^28^ have reported that *DDX23* has a role in promoting the malignancy of gliomas in the brain. It is intriguing to speculate whether *DDX23* was associated with both BPP and rhabdomyosarcoma in the young female FINLIS18-3 in this study. Interestingly, PMG has also been reported in patients with pathogenic variants in another *helicase* genes *DHX37*^18^ and *DDX3X.*^31^

Finally, despite incorporating various genomic techniques alongside standard-coverage ES, the diagnostic yield of BPP in this investigation aligns closely with findings from prior studies. However, it remains notably lower than diagnostic rates for other congenital brain malformations, such as lissencephaly, in which the diagnostic rate is ∼80%.^32^ This discrepancy may be attributed to an increase in somatic variants that are exclusively present in a few or single tissues. In addition, the variant may have a very low-level mosaic in the tissues studied here (blood/buccal) and therefore may need a deeper analysis than 200×. To enhance the identification of causal variants in these disorders, there is a pressing need for the adoption of more sophisticated, preferably minimally invasive sampling techniques and sequencing methodologies.

In summary, our data suggest an increased incidence of phenotypic and genotypic heterogeneity of BPP and call for the use of new methods to elucidate the biological background of BPP.

## Supplementary Material

fcae142_Supplementary_Data

## Data Availability

Variants have been deposited into ClinVar under accession numbers SCV004171801–SCV004171806.
