# Supplementary material for: Heterogeneous genetic patterns in bilateral perisylvian polymicrogyria: insights from a Finnish family cohort
Source: Brain Commun. 2024 Apr 18;6(3):fcae142. doi: 10.1093/braincomms/fcae142 (PMC11073749; doi:10.1093/braincomms/fcae142)
Supplement: fcae142_Supplementary_Data [file fcae142_supplementary_data.docx]

**SUPPLEMENTAL MATERIALS**

**Clinical description of the families**

**FINLIS1-3** is a 34-year-old male, first child of the family, and second child of the mother. The mother and the index case have deafness. Hearing impairment was noticed at 5 years in the mother and at 3 years in FINLIS1-3. Deafness developed during the next 5-10 years. Pregnancy and delivery were normal. Delay in psychomotor development was detected soon after birth. Reflex seizures started from the age of 3 years, which were provoked by drinking. The seizures included head drops and later, focal-onset tonic seizures, the latter continuing into adulthood.

A hearing aid was fitted at 3 years-of-age, but the proband never developed speech, but he communicates in sign language. FINLIS1-3 learnt to read and write at 5-6 years. His full-scale IQ was borderline (WISC-R). He attended a special school for the hearing impaired and completed a 3-year education in applied sciences. He is living in a flat with services for deaf people.

He had mild right-dominant pyramidal signs, slow fine finger movements, and lower face and tongue paresis, an abnormally brisk jaw jerk, and decreased movement of the soft palate. He had some sialorrhea. Emotional facial expressions were normal. MRI of the brain showed widespread bilateral fronto-parietal and perisylvian-insular polymicrogyria. The mother had normal brain morphology on MRI.

In FINLIS1-3, loud auditory stimulation elicited normal evoked responses in the left auditory cortex. A likely pathogenic (LP) missense variant c.923C>G:p.(S308C) in *WFS1* was identified in the affected son and his mother explaining their hearing impairment.

The son also has a novel hemizygous missense variant p.(S1068L) in *Alf transcription elongation factor 2* (*AFF2) (*OMIM # 309548) that was classified as a variant of unknown significance (VUS)*.* His mother is an unaffected carrier of the variant.

**FINLIS2-3** is an 18-year-old male, the first of two children in the family. He was born using forceps after a 37-week pregnancy. He has had profuse sialorrhea from early infancy and his mouth was continuously open. He learnt to walk at the age of 15 months, said his first words at 28 months, and first sentences at 3.5 years-of-age (vowel speech). Severe oral motor difficulties were diagnosed: absent side-to-side tongue movements and weak lower face movements. He has myopia. From the age of 6 months, he suffered repeated episodes of vomiting and lethargy for 2 years. At 3.5 years, both verbal and nonverbal cognitive skills were considered normal, except for speech expression and hyperactivity, and problems with sustained attention. At 15 years, he started to have tonic-clonic seizures. He is currently seizure-free on anti-epileptic medication (valproate and oxcarbazepine). A new assessment at the age of 15 years, suggested mild intellectual disability (ID). He has completed professional education and is employed as a janitor. FINLIS2-3 has good social skills. Sialorrhea, eating, communication, as well as attentional challenges are his main problems in young adulthood. Brain MRI showed BPP.

Exome sequencing (ES) revealed a *de novo* pathogenic variant [c.4862G>A:p.(R1621Q)] in *SCN3A*.

**FINLIS 3-3** is a 31-year-old female, first of six children in the family. She was born after a normal pregnancy and delivery. Her birth weight was 3980 grams (g), and length 51 centimeters (cm). She learnt to walk at 24 months and started to sign words at 48 months. She was diagnosed with mild left-sided hemiparesis and anarthria, as well as dysphasia and mild intellectual impairment. She attended a special class for pupils with language and hearing impairment.

When seen at the age 11 years, symmetric mild weakness of the lips was noted, as well as missing lateral tongue movements left of the midline, but some movement was present to the right. Muscle tone and reflexes were normal, and her plantar response extensor as well. Median nerve stimulation gave normal evoked responses in the contralateral polymicrogyric sensorimotor cortex. Her communication is mainly challenged by special language impairment, and less by articulation problem. One epileptic seizure occurred at the age of 3 years, and a second seizure at the age of 10 years. At 11 years, multi-focal epileptic spikes were generated in the polymicrogyric cortex. She is currently using anti-epileptic medication. MRI of FINLIS3-3 shows bilateral perisylvian and perirolandic polymicrogyria, which is more extensive in the right hemisphere.

No relevant variant was obtained in ES.

**FINLIS4-3** is **a** 22-year-old female, the third of seven children in the family. Her mother was operated on for Fallot’s tetralogy during childhood. FINLIS4-3 was born after a normal pregnancy and delivery with a birth weight of 3580 g, length of 50 cm, and head circumference of 34.5 cm. Breastfeeding (sucking) was successful, but problems with swallowing were suspected. Profuse sialorrhea was present from her early weeks and later she had problems with chewing food. She was unable to move her tongue from side to side. She learnt to walk at 19 months. Brief absences were suspected at the age of 22 months. An EEG showed bilateral multifocal spike-waves in her sleep. At 24 months, nocturnal epileptic myoclonus was treated with clobazam, and no seizures were detected until school age, when valproate was started**.** At 2 years-of-age, very mild right-dominant tetraplegia was diagnosed. At 5 years-of-age, mild ID was diagnosed. She communicates in sign language and uses a tablet for writing and as a speech synthesizer. MRI at 22 months showed bilateral perisylvian and perirolandic polymicrogyria.

ES revealed a *de novo* pathogenic missense variant [c.776C>T:p.(P259L)] in *TUBB2B* (OMIM #610031).

**FINLIS5-3** is a 24-year-old female, and an only child. Pregnancy and delivery were normal. At birth, she got an Erb’s paresis of her left arm. Her birth weight was 4520 g, length 53.5 cm, and head circumference 35.5 cm. She has sialorrhea and problems with chewing solid foods. She learnt to walk at 6 years-of-age. She had a combination of left-predominant central quadriplegia and some degree of peripheral nerve lesion. She communicates with gestures, vocalization, mimics, and uses a synthesizing device. Her tongue movements are limited to extension-retraction. Her oral cavity and lower face are hypersensitive. Epileptic seizures (atypical absences) started around 20 months-of-age and were provoked by sensory stimulation of the face and mouth. Subtotal callosotomy at the age of 4 years stopped her seizures, and she has been seizure-free for years on the antiepileptic drugs etosuximide and topiramate. At 6 years-of-age, moderate to severe ID was diagnosed. At 24 years, her social skills are normal and she has many friends. Her MRI showed wide-spread peri-sylvian and perirolandic polymicrogyria.

ES and optical genome mapping (OGM) gave no relevant variant.

**FINLIS 6-3** is a 28-year-old female, the first of two children. Pregnancy and delivery were normal. Birth weight was 3420 g, length 48 cm and head circumference 33.5 cm. Breastfeeding was terminated due to choking. She learnt to walk at 10 months-of-age, and to speak at 3 years. Her speech is dysarthric. She coughed easily during meals. Her oral-motor functions were very challenging, with profuse sialorrhea. Absent side-to-side movements of the tongue prevented her from chewing so a gastrostomy was performed. She had mild pyramidal signs on the left side of her body. Her fine finger movements are compromised by a tremor. Therapy resistant epileptic seizures (atypical absences) started at the age of 3 years and were treated using early adrenocorticotropic hormone (ACTH) -therapy and anterior callosotomy. Interestingly, her seizures spontaneously stopped at 14 years-of-age. She has moderate ID. She currently lives in a family with other intellectually disabled youths and attends regular supported work activities. MRI found bilateral perisylvian/perirolandic polymicrogyria.

No relevant variant was found in ES and OGM.

**FINLIS 7-3** is a 31-year-old male, the second of two children. First extra-uterine pregnancy ended with abortion. His mother, FINLIS7-2 has one healthy brother and two stillborn siblings, one of which had hydrocephalus. The mother of FINLIS7-2 has a twin brother with mild ID. FINLIS 7-3 had a birth weight of 3160 g, length of 51 cm, and head circumference of 36.5 cm. His growth slowed down to the 2.5% percentile [-2 standard deviations (SD)] during the first 2 years. He could not suck from the breast but managed to suck from a bottle. He learnt to walk at 4.5 years-of-age. He communicates via sign language. Hypogonadism, horse-shoe kidney, umbilical hernia, and bilateral inguinal herniations were detected after birth. His facial features included low-set ears and hairline, hairy areas partly covering the cheeks in front of both earlobes, synophrys, stenosis of the right lacrimal duct, high-arched narrow palate, long philtrum, small nose, anteverted nostrils, strabismus, and enamel hypoplasia. At 3 years, bilateral optic atrophy and uneven retinal pigmentation were discovered. He had hip luxation during infancy, club foot on the right, bilateral short metacarpal and metatarsal bones, bilateral ulnar deviation of 4^th^ and 5^th^ fingers, palmar crease, and partial syndactyly of toes. He has moderate to severe ID. He lives in a supported community for the intellectually disabled. His MRI shows bilateral but right-hemisphere-dominant fronto-opercular polymicrogyria. Prometaphase chromosomes, thyroid function, urinary screen for amino acids, oligosaccharides and gangliosides were normal.

No relevant variant was found in ES and OGM.

**FINLIS8-3**is a 27-year-old male, the second of three children. His birth weight was 2535 g and length 47 cm. His right-hand fingers and right-leg toes had multiple distal amputations diagnosed as a result of amniotic band syndrome. As a newborn, his duodenal atresia was operated on. He had sialorrhea and substantial feeding problems.

FINLIS8-3 learnt to speak at 5-6 years of age. The speech is unclear. He has esotropia in the right eye. The movement of palatal arches were slow and reduced. He has borderline ID. He received special education and conducted studies in applied sciences. He is the father of a 6-year-old daughter.

MRI revealed BPP with slightly large lateral ventricles, absent septum pellucidum, bilaterally wide Sylvian fissures, and a thick polymicrogyric cortex lining that extends to the posterior part of lateral ventricles.

ES and OGM were negative. Deep ES revealed a heterozygous VUS splice region variant c.3531-4A>G in *DOCK8*.

**FINLIS9** is a 43-year-old female whose parents are unknown. Oral-motor difficulty was severe with absent lateral movements of the tongue during vocal speech. Her profuse sialorrhea was reduced after the removal of her salivation glands at the age of 7 years. She learnt to walk at 1 year of age, was right-handed with strong contra-lateral mirror movements of her left hand. She had focal epilepsy with right-hemisphere-onset spike-wave discharges between 3 - 12 years of age. She has normal intelligence, is married, and is the mother of three children. Her brain MRI showed slightly asymmetric right-dominant perirolandic and perisylvian polymicrogyria.

No relevant variant was found in ES.

**FINLIS10-3** is a 27-year-old female, third of a three children of her family. During pregnancy, her mother was treated with aspirin against SLE systemic lupus erythematosis. Her birth weight was 3270 g and length 50 cm. Her length and head circumference followed the – 2SD (2.5%) percentile and the length-related weight the -10% percentile. As a newborn she choked often, followed later by difficulties in chewing and eating solid foods. She could move her tongue towards the right, but not towards the left of the midline. Speech production was difficult, but language understanding was normal. She spoke single words from the age of 1 year, and she used several word sentences in signs language at 2 years. She learnt to walk 2-3 years of age. She has spastic diplegia with some spasticity evident also in her finger movements. Her social skills were normal. Her speech production remained challenging with vocal speech. Visuo-motor skills were considered normal. She lives independently, has a driving license, and participates in activities appropriate for her professional education as a visual communication designer. Her brain MRI at 1 year revealed BPP.

No relevant variant was found in ES and OGM.

**FINLIS11-3** is a 35-year-old male, the second child of two siblings. His birth weight was 4200 g, and length 54.5 cm. The right arm was floppy during the neonatal period. He learnt to walk at 16 months-of-age. At the age of 10 years, his fine- and gross-motor skills were clumsy. He has severely reduced voluntary movements of the mouth and tongue, and very mild right-dominant quadriplegia. Due to the absent or reduced voluntary movements of tongue and lower face, articulation and eating were challenging. Language and cognitive development were normal. He did progress well in a special school with a mainstream educational program. He had no signs of epilepsy. His brain MRI revealed BPP.

No relevant variant was found in ES.

**FINLIS12-3** was born as the first of 3 children. Father, FINLIS12-1, experienced two epileptic seizures during his army service. During pregnancy, FINLIS12-3’s mother had hypothyreosis treated with thyroxin. FINLIS12-3 was born at term with a birth weight of 3470 g. Breastfeeding was unsuccessful. His phenotype was characterized by severe oral motor problems; anarthria and prominent sialorrhea, as well as hypersensitivity of the lower face and oral cavity that made chewing, tongue movements, and oral speech impossible. His tongue had continuous involuntary sucking movements. He learnt to walk at 14 months. He communicated mainly with signs. He had a mild ID. Epileptic seizures started at 10 years-of-age but occurred infrequently. At the age of 18 years, he drowned due to a seizure while swimming. His brain MRI revealed BPP as well as extensive perisylvian and fronto-parietal polymicrogyria.

**FINLIS12-1** is a 65-year-old father of FIN12-3, who had two epileptic seizures while being in the army. He was treated with sodium channel blockers for 5 years and has not had further seizures. One of his maternal female cousins, and the daughter of another maternal cousin also have had epilepsy. The MRI of FINLIS12-1 revealed a large area of polymicrogyric-like heterotopia within the white matter of the right temporal lobe.

In ES a novel Autosomal Dominant VUS variant c.985C>T:p.(Q329*) of *GPR37L1* was identified in both father and son.

**FINLIS13-3** is a 30-year-old female, the third child of three siblings with parents of Roma origin. Her birth weight was 3450g, length 50.5 cm, and head circumference 32 cm (-2.5 SD). After birth, gr. II-III systolic heart murmur were detected. A trabeculated right ventricle was found that normalized by 7 months-of-age. She learnt to walk at 2 years and 7 months-of-age. Her adult length is 150 cm and head circumference 50 cm (-2 SD). She has left-dominant spastic tetraplegia. She lacks horizontal tongue movements to the left. Her speech is fluent but unclear. She sometimes chokes while swallowing. She has mild to moderate ID. She uses a tablet for writing. Her fine motor skills do not permit readable handwriting. A brain MRI revealed abnormal gyration in the frontal and parietal lobes, and substantially reduced parietal-lobe volume.

In ES an Autosomal Recessive homozygous VUS variant c.787G>A:p.(D263N) in coiled-coil domain-containing protein 82 (*CCDC82)* (OMIM #619870) was identified. Both parents were heterozygous for the variant.

**FINLIS14-3** is a 14-year-old female, the first of two children. The first year development was normal except for profuse sialorrhea. A severe sensorineural hearing impairment was diagnosed at the age of 2 years. Cochlear implants were inserted to the right ear at 30 months and to the left ear at 60 months of age. Her speech is unclear, and she prefers signs to communicate. She can close her lips and tilt her head back to avoid sialorrhea. Oral motor function is compromised by unusual sensitivity of the oral cavity. She learnt to walk at 20 months-of-age. Her fine motor skills were better in the left than the right hand. She is unable to move the 4th and 5th fingers of her right hand voluntarily, which makes her sign language clumsy. She attends a school for hearing impaired children using a personal training program. Neuropsychological assessment at the age of 14 years revealed mild ID. Her social skills are good, but her daily activities are challenged by considerable anxiety in unfamiliar situations. MRI showed BPP and bilateral white matter changes around the posterior horns of lateral ventricles.

A *de novo* likely pathogenic variant c.616G>T:p.(D206Y) in *NUS1* was found in ES.

**FINLIS15-3** is a 36-year-old male, and the only child of the family. When seen at the age of 13 years, he was independently walking, dysarthric, and could write his name in block letters. No hospital records are available.

A novel VUS missense variant c.2251A>G:p.(I751V) in *BOC* was identified in ES.

**FINLIS16-3** is 25-year-old male, the third of 4 children, and he has a twin brother. He was born at 33 weeks of gestation. His twin brother is healthy. FINLIS16-3 had birth weight of 1780 g. His facial features are characterized by dolichocephaly, strabismus, and prominent maxilla and teeth. As an infant over 100 hemangiomas sized 2-3 mm were detected which disappeared by age. At the same age, infantile spasms were diagnosed, and he continued to have severe epilepsy. He cannot speak or walk. He has autistic features, spasticity and dystonia. His brain MRI shows BPP extending to the frontal lobes. Migration panel, molecular karyotype, metabolic and mitochondrial investigations were normal.

ES and OGM showed no relevant variants.

**FINLIS18-3** is 4^th^ child of the unaffected parents. The three older sisters are unaffected. During pregnancy, fetal movements were reduced, and the amount of amniotic fluid was increased. FINLIS18-3 was breach delivered at term without asphyxia. Her birth weight was 2850 g (-1.8 SD), length 46 cm (-2SD), head circumference 36.5 cm (+1.5 SD). Her length at 15 years was 156 cm (-1.8 SD). At birth, she was diagnosed with Pierre-Robin sequence and arthrogryposis on multiple joints. Big problems of swallowing with aspiration led to tube feeding from day 1, intubation from day 5, and permanent tracheostomy at the age of one month. Permanent gastrostomy for feeding was inserted at 2.5 years.

Vocal cords moved symmetrically, and the coughing reflex was preserved. The lower jaw, the tongue and the epiglottis were hypoplastic. There was no gag reflex, the upper esophageal sphincter, the tongue and other pharyngeal muscles were floppy preventing swallowing, but not aspiration or esophageal reflux. At 1 year of age, involuntary tremor was seen in the tongue and in the soft palate. Voluntary facial nerve functions were weak, the mouth was open, and only small voluntary protrusion of the tongue and of the lips were successful. Emotional smile was preserved. Hypersensitivity to touch of lips and lower face was suspected, but sensitivity in the oral cavity was considered normal. She could bite with the back teeth but was unable to process food or swallow. She never learnt to produce any vocalization.

Vision, eye movements, the corneal reflex and hearing were normal. Tendon reflexes in the right leg were normal, and difficult to elicit in stiff extremities. She underwent several orthopedic operations and year-long occupational- and physiotherapy to help locomotion and fine motor skills. She was able to stand up at 1.4 year and walk unsupported from the age of 3 years. Her walking distance at school age was close to 1 kilometer, and she used wheelchair or sticks on longer distances. She needed life-long help in most daily activities (dressing, toileting, feeding, tracheostoma maintenance).

Language understanding, verbal and nonverbal IQ were age appropriate before school. On the 1^st^ grade, her neuropsychological profile showed normal performance. With special education, she completed her basic education with average records. She used sign language and gestures for communication, where it was understood, cellphone SMS with her peers, and pc for schoolwork. Excessive sialorrhea was socially the most disturbing symptom in school age.

Slightly before her 17^th^ birthday, an aggressive rhabdomyosarcoma appeared in the left cheek. The tumor turned out resistant to radiation and chemotherapies, and invaded large areas of the neck and extra-cranial structures, and finally the left hemisphere. Brain MRI at ages 4 and 8 years showed BPP.

Further etiological investigations revealed normal muscle biopsy and electric muscle activity, normal heart and abdominal structures on several ultrasound studies. No immunological signs of prenatal viral infection was detected. Karyotype was normal, and the CATCH22 microdeletion was excluded. The patient deceased at the age of 19 years.

ES revealed a *de novo* likely pathogenic variant in *DDX23* [c.2437C>T:p.(R813C)].

**FINLIS19-3** is a 5-year-old male, the only child of the parents. Asymmetric motor development with right-side delay was noticed during infancy. He learnt to walk at 15 months. He has severely reduced voluntary movements of the mouth and tongue, right-hand clumsiness, epilepsy. He did not learn to speak and uses signs to make himself understood. Shortly before the age of 2 years, he was diagnosed with epilepsy. EEG showed continuous bilateral spike-waves in his sleep. He has learning problems and has moderate developmental delay. BPP was present in the brain MRI. His molecular karyotype and migration panel were normal.

Deep ES and OGM did not show any relevant variants.

**FINLIS20-3** is 3-year-old male and the only child of the family. He was born after an uneventful 35-week pregnancy. His birth weight was 2504g, length 48 cm, and head circumference 31.5 cm. As a newborn, oral motor dysfunction was found. He can bite but cannot chew properly. He has had profuse sialorrhea since infancy. He learnt to walk at 2.5 years-of-age. FINLI20-3 said several words at 3 years-of-age. He also communicates by pointing and by simple signs. He has mild left-dominant tetraparesis. He has delayed fine motor skills. He has some spike-wave activity in EEG. The brain MRI showed perisylvian polymicrogyria. Molecular karyotype, neuronal migration disorders panel* CeGat (Tübingen, GmbH) is normal.

Deep ES and OGM were negative.

**FINLIS21-3** is an 11-year-old male, first child of two siblings. His birth weight was 3375 g, length 50 cm, and head circumference 34.5 cm. As a newborn, he coughed when breastfed. Asymmetric motor and fine motor development with left-side delay was noticed during infancy.  He learnt to walk at 13 months. Mouth motor functions are challenging, with profuse drooling. Despite obvious dysarthria, his language development was not abnormal: as an infant, he babbled a lot, said his first words at 1 year and first sentences at 3 years-of-age. He has some difficulties with his attention span. In reduced-size main-stream classes he makes average progress. His brain MRI revealed BPP. His molecular karyotype shows a 163 kb deletion in the 7q31.1 region considered as a VUS.

In ES a hemizygous X-linked VUS variant c.4640A>G:p.(N1547S) was found in the *TAF1* gene. The mother is a non-affected carrier of the variant.

**FINLIS22-3** is a 9-year-old female, the first of two children of the family. Her birth weight was 3730g, length 50 cm, and head circumference 35 cm. At birth, asymmetry of the eyes was noticed with the right palpebral fissure appearing smaller than the left, and it could not completely close. There was a slight corneal opacity on the left eye. She had a strong strabismus and a head tilt. From the age of 2 years, she was using sunglasses to prevent sunlight-induced attacks of vomiting and headaches, interpreted as migraines. At the age of 17 months, spastic diplegia was diagnosed. She learnt to walk at the age of 5 years. By 9 years, her maximum walking distance is 50 meters, and she uses a walking aid (rollator) during daily life, and wheelchair for long distances. She is left-handed, holds a spoon in her fist and her fine motor skills are clumsy. Due to delayed language development, speech therapy was started at the age of 3 years. She had no significant oral motor problems with articulation, chewing or swallowing. Her tongue movements are normal. At 5 years-of-age, she performed at the level of a 2.5-year-old according to neuropsychological tests, so she attended a special class with individual support. She has learnt to read, write, and calculate. Her main challenges are impulsivity, attention deficit, and poor judgement. Her MRI shows BPP, mainly in the insular and posterior parietal opercular cortex.

A pathogenic *de novo* missense variant c.303T>A:p.(N101K) in *TUBA1A* was identified using ES.

**Supplemental table 1. Summary of phenotypic features and methods used in this cohort**

| **Patient #** | **Gender** | **Age** | **Exome (blood)** | **Deep exome (buccal)** | **OGM** | **Severity of ID or DD** | **MRI/CT** | **Other features** |
| --- | --- | --- | --- | --- | --- | --- | --- | --- |
| FINLIS1 | M | 34 | two disorders |  |  | normal | BPP | Wolfram -like syndrome |
| FINLIS2 | M | 18 | positive |  |  | mild | BPP | epilepsy |
| FINLIS3 | F | 31 | negative |  |  | mild | BPP |  |
| FINLIS4 | F | 22 | positive |  |  | mild | BPP | epilepsy |
| FINLIS5 | F | 24 | negative | negative | negative | moderate | BPP | severe epilepsy |
| FINLIS6 | F | 28 | negative | negative |  | moderate | BPP | severe epilepsy |
| FINLIS7 | M | 31 | negative | negative | negative | moderate-severe | BPP | dysmorphic facial features |
| FINLIS8 | M | 27 | negative | inconclusive | negative | borderline | BPP | amniotic band syndrome |
| FINLIS9 | F | 43 | negative |  |  | normal | BPP |  |
| FINLIS10 | F | 27 | negative | negative | negative | normal | BPP |  |
| FINLIS11 | M | 35 | negative |  |  | normal | BPP |  |
| FINLIS12 | M | 37 | inconclusive |  | negative | mild-moderate | BPP | epilepsy |
| FINLIS13 | F | 30 | inconclusive |  |  | mild | BPP | microcephaly |
| FINLIS14 | F | 14 | positive |  |  | mild | BPP | hearing impairment |
| FINLIS15 | M | 35 | inconclusive |  |  | ND | BPP |  |
| FINLIS16 | M | 19 | negative | negative | negative | severe | BPP |  |
| FINLIS18 | F | 19Ϯ | positive |  |  | ND | BPP | rhabdomyosarcoma |
| FINLIS19 | M | 6 | negative | negative | negative | mild | BPP | epilepsy |
| FINLIS20 | M | 3 | negative | negative | negative | too young | BPP | epilepsy |
| FINLIS21 | M | 11 | inconclusive | negative |  | normal | BPP | dysmorphic facial features |
| FINLIS22 | F | 11 | positive |  |  | moderate | BPP | spastic diplegia |

**Supplemental table 2. Identification of variants of unknown significance in the study cohort**

|  | | | |  |  |  |  |  |  |
| --- | --- | --- | --- | --- | --- | --- | --- | --- | --- |
| **Patient *#*** | **Sex** | **Age** | ***Gene*** | **Variant** | **Inheritance** | **Severity of ID or DD** | **MRI/CT** | **Other features** | **ACMG** |
| FINLIS1 | M | 34 | *AFF2* | NM_002025.4:c.3203C>T:p.(S1068L) | XLR | borderline | BPP |  | VUS |
| FINLIS8 | M | 27 | *DOCK8^a^* | NM_203447.4:c.3531-4A>G | heterozygous (absent from one parent) | borderline | BPP | amniotic band syndrome | VUS |
| FINLIS12 | M | 37 | *GPR37L1* | NM_004767.5:c.985C>T:p.(Q329*) | AD (inherited from affected parent) | mild-moderate | BPP | epilepsy | VUS |
| FINLIS13 | F | 30 | *CCDC82* | NM_024725.4:c.787G>A:p.(D263N) | AR (homozygous) | mild | BPP | microcephaly | VUS |
| FINLIS15 | M | 35 | *BOC* | NM_001301861.2:c.2251A>G:p.(I751V) | heterozygous (absent from one parent) | ND | BPP |  | VUS |
| FINLIS21 | M | 11 | *TAF1* | NM_004606.5:c.4640A>G:p.(N1547S) | XLR | normal | BPP | dysmorphic facial features | VUS |

^a^Identified via deep exome sequencing
